# Metronomic combination of Vinorelbine and 5Fluorouracil is able to inhibit triple-negative breast cancer cells. Results from the proof-of-concept VICTOR-0 study

**DOI:** 10.18632/oncotarget.25422

**Published:** 2018-06-08

**Authors:** Maria Grazia Cerrito, Marco De Giorgi, Davide Pelizzoni, Sara Maria Bonomo, Nunzio Digiacomo, Arianna Scagliotti, Cristina Bugarin, Giuseppe Gaipa, Emanuela Grassilli, Marialuisa Lavitrano, Roberto Giovannoni, Paolo Bidoli, Marina Elena Cazzaniga

**Affiliations:** ^1^ Department of Medicine and Surgery, University of Milano-Bicocca, Monza 20900, Italy; ^2^ Oncology Unit, ASST Monza, Monza 20900, Italy; ^3^ Phase 1 Research Centre, Monza 20900, Italy; ^4^ M.Tettamanti Research Center, Pediatric Clinic, University of Milano Bicocca, Monza 20900, Italy

**Keywords:** triple-negative breast cancer, 5-fluorouracil, vinorelbine, metronomic combination, LC3A/B

## Abstract

Triple Negative Breast Cancer (TNBC) is an aggressive neoplasia with median Overall Survival (OS) less than two years. Despite the availability of new drugs, the chance of survival of these patients did not increase. The combination of low doses of drugs in a metronomic schedule showed efficacy in clinical trials, exhibiting an anti-proliferative and anti-tumour activity. In Victor-2 study we recently evaluated a new metronomic combination (mCHT) of Capecitabine (CAPE) and Vinorelbine (VNR) in breast cancer patients showing a disease control rate with a median Progression-Free Survival (PFS) of 4.7 months in 28 TNBC patients.

Here in Victor-0 study, we examined the effect of mCHT vs standard (STD) schedule of administration of different combinations of 5-Fluorouracil (5FU), the active metabolite of CAPE, and VNR in TNBC cell lines MDA-MB-231 and BT-549. A significant anti-proliferative activity was observed in cells treated with metronomic vs STD administration of 5FU or VNR alone. Combination of the two drugs showed an additive inhibitor effect on cell growth in both cell lines. Moreover, after exposure of cells to 5FU and VNR under mCHT or conventional schedule of administration we also observed a downregulation of chemoresistance factor Bcl-2, changes in pro-apoptotic protein Bax and in cleaved effector caspase-3 and increased expression of LC3A/B autophagy protein.

Our results therefore suggest that molecular mechanisms implicated in apoptosis and autophagy as well as the cross-talk between these two forms of cell death in MDA-MB-231 and BT-549 cells treated with 5FU and VNR is dose- and schedule-dependent and provide some insights about the roles of autophagy and senescence in 5FU/VNR-induced cell death.

## INTRODUCTION

TNBCs are a specific subtype of epithelial breast tumors that are immunohistochemically negative for the protein expression of estrogen receptor (ER), progesterone receptor (PR) and do not show overexpression/gene amplification of HER2 [1].

TNBCs account for about 10–20% of all breast cancers and are associated with a very bad prognosis, even in early stages of disease: after radical surgery, median time to relapse is approximately 18 months and median OS is less than 24 months. Despite the big efforts aiming to improve this clinical scenario and to understand the molecular basis of breast cancer biology little has really changed in the last decades for these patients [2].

Rest periods between two consecutive cycles of chemotherapy administered at Maximum Tolerated Dose (MTD) is necessary to allow recovery from toxicities. Unfortunately, there is evidence not only of re-growth of tumour cells but also of growth of selected drug-resistant clones [3]. To improve the therapeutic index of chemotherapy it is necessary to modify the choice of drugs or to change the way of administration. In this scenario, mCHT-which refers to regular admistration of conventional chemotherapy drugs at low, minimally toxic doses, with no prolonged break periods [4]-could represent a promising therapeutic option for advanced breast cancer patient. Recently, it has been shown that mCHT has an important stabilizing effect on cancer growth (including chemotherapy-resistant disease) and confers prolonged clinical benefits by improving at the same time the quality of life of cancer patients by avoiding severe toxicity [5–8].

Likewise, many studies have demonstrated that the tumor response to metronomic schedules is due both to antiangiogenic/immuno-stimulatory effects and to direct effects on tumor cells themselves. Therefore, mCHT can be defined as a multitargeted therapy, able to strike both tumor cells and the surrounding microenvironment [9].

Different authors [10, 11] have explored the use of mCHT in TNBC patients, reporting a wide range of Overall Response Rate (ORR, 9–44%) and median PFS of approximately 10 months.

Metronomic combination of VNR and CAPE has been recently studied in 80 advanced breast cancer patients [7], of whom 28 were TNBC, suggesting a promising activity in terms of Clinical Benefit Rate and PFS.

All these results observed in the clinical practice are little supported by pre-clinical data, mainly for what concerns the combination of different agents, all given in a metronomic way.

In order to identify and describe which kind of biological processes are implicated in determining the results observed in the clinical practice here, we evaluated the antiproliferative and cytotoxicity effects of 5FU and/or VNR given either in STD or in metronomic schedule, on MDA-MB-231 and BT-549 cells.

## RESULTS

### Metronomic administration of 5FU and VNR significantly inhibits human MDA-MB-231 and BT-549 breast cancer cells growth

The effect on cell viability of 5FU and VNR, alone or in combination, either given in STD or in mCHT administration, was investigated using the MTT assay. For these studies, MDA-MB-231 and BT-549 cells were treated with 5FU or VNR at the indicated dose, for 4 or 96 hours to simulate the conventional (4 h) or metronomic (96 h) dosing protocol.

**Figure 1:**
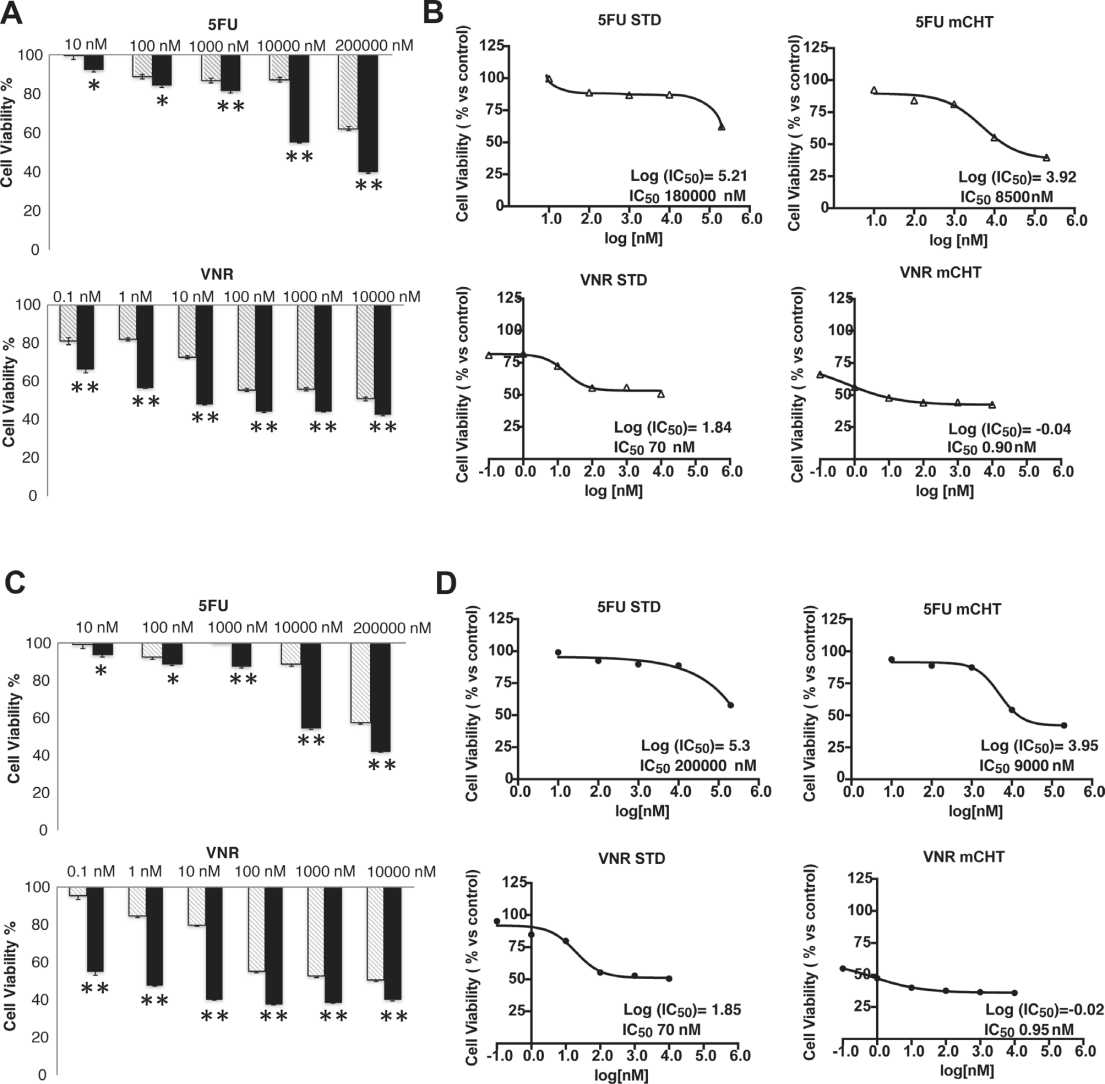
Metronomic administration of 5FU and VNR induced significant growth inhibition in human MDA-MB-231 and BT-549 breast cancer cells (**A**) MDA-MB-231 and (**B**) BT-549 cells were treated with different concentration of 5FU and VNR for 4 h (STD) or 96 h (mCHT). The dose-response curves of MDA-MB-231 (**C**) and BT-549 (**D**) were used to calculated IC_50_ value. Cell viability was investigated using the MTT assay and expressed as a percentage relative to the untreated control cells. The experiment was repeated 4 times with at least 8 replicates per sample. ^*^p < 0.05 *vs* untreated ^**^p < 0.01 *vs* untreated. Results are means ± SD of three measurements (*P <* 0.05).

A significant anti-proliferative activity was observed in both cell lines treated with metronomic administration of 5FU or VNR compared to STD treatment (Figure 1A, 1C).

**Figure 2:**
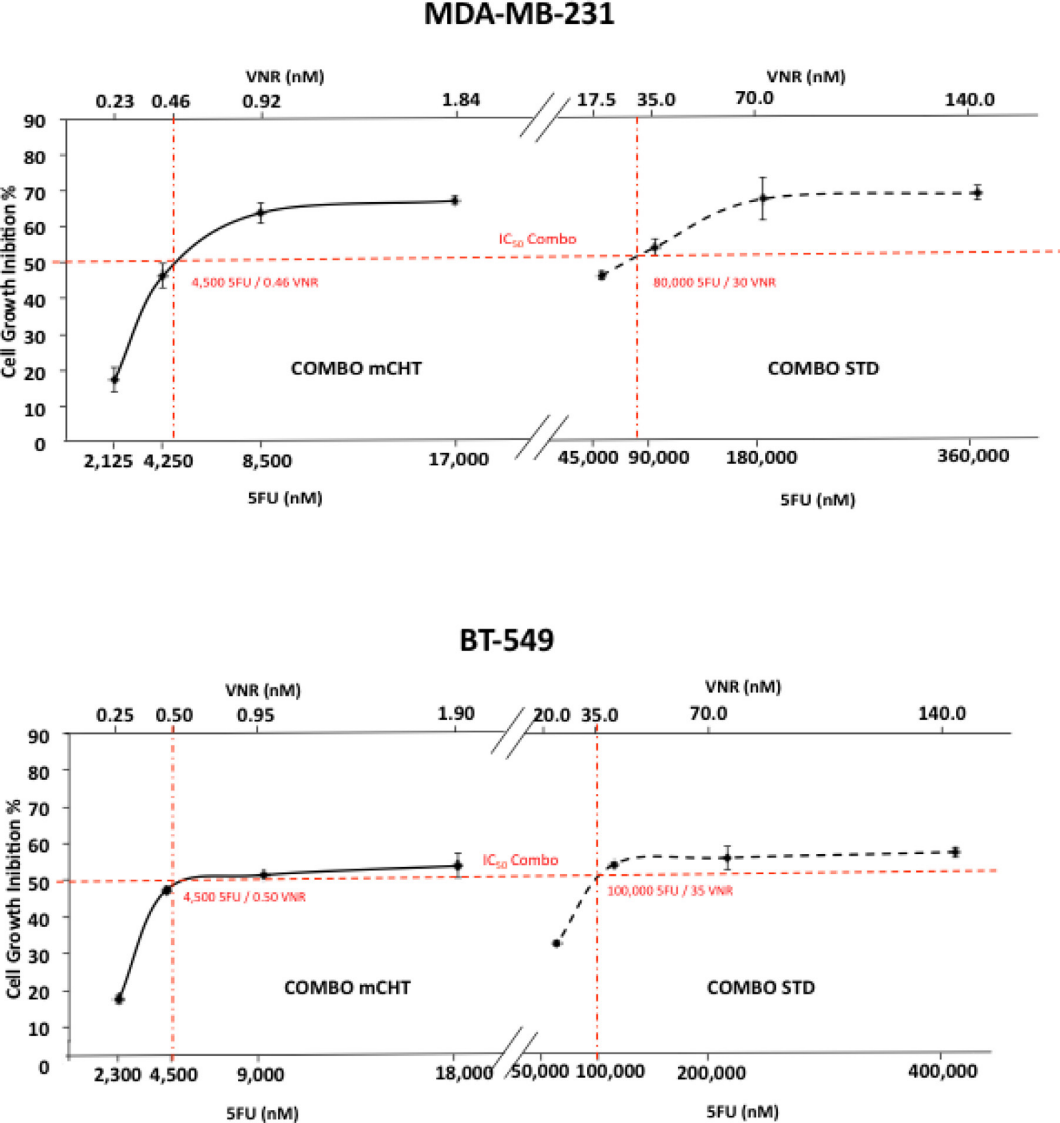
Metronomic administration of 5FU and VNR in combination induced significant growth inhibition in human MDA-MB-231 and BT-549 breast cancer cells Representative dose-response curve performed on MDA-MB-231 (**A**) and BT-549 (**B**) treated with the following drug combination: 1) 2x IC_50_ (5FU) + 2x IC_50_ (VNR) 2) IC_50_ (5FU) + IC_50_ (VNR) 3) ½ IC_50_ (5FU) + ½ IC_50_ (VNR); 4) ¼ IC_50_ (5FU) + ¼ IC_50_ (VNR); cells were treated for 4 h (STD) or 96 h (mCHT) and their number evaluated by MTT assay. The reading values were converted to the percentage and compared to untreated control. The simple two-point method uses 2 data points bracketing 50% inhibition of proliferation (red lines) to estimate the IC_50_. The experiment was repeated 3 times with at least 8 replicates per sample.

Concentrations of drugs provoking 50% cell growth inhibition (IC_50_) were calculated from curves derived by plotting cell viability (%) versus drug concentration (nM). The reading values were converted to the percentage of the control. The IC_50_ of single-dose of 5FU administration with the metronomic schedule was more than 20 times lower in both cell lines compared to standard treatment (MDA-MB-231:8500 nM *vs* 180000 nM; BT-549: 9000 nM *vs* 200000 nM). The IC_50_ of VNR at 96 h was a couple orders of magnitude lower in MDA-MB-231 and BT-549 cells treated with the metronomic schedule in comparison to the exposure with VNR at conventional concentrations (0.92 nM *vs* 70 nM and 0.95 nM *vs* 70 nM respectively) (Figure 1B and 1D, Table 1). The combination ratio was calculated using the IC_50_ ratio of the single drugs, so that the contribution of the effect for each drug in the mixture would be the same. The results are summarized in Figure 2 which shows the combination index (CI) of the IC_50_. Synergistic, additive, or antagonistic effects were defined using the CI method of Chou-Talalay [12]. As shown in Table 1, co-incubation of 5FU with VNR showed additive effects on both cell lines with CI values in the range of 0.9 and 1.0.

**Table 1:** 5FU and VNR concentrations used for combination treatment of MDA-MB-231 and BT-549 cells in the STD and mCHT schedule

		IC_50_ Single treatment		IC_50_ Combo treatment		Chou index CI = (D_1_/Dx_1_) + (D_2_/Dx_2_)
**MDA-MB-231**	**STD**	**5FU**	180000 nM	**5FU+VNR**	80000 nM + 30 nM	**0.9**
		**VNR**	70 nM			
	**mCHT**	**5FU**	8500 nM	**5FU+VNR**	4500 nM + 0.5 nM	**1**
		**VNR**	0.92 nM			
**BT-549**	**STD**	**5FU**	200000 nM	**5FU+VNR**	100000 nM + 35 nM	**1**
		**VNR**	70 nM			
	**mCHT**	**5FU**	9000 nM	**5FU+VNR**	4500 nM + 0.50 nM	**1**
		**VNR**	0.95 nM			

IC_50_ value for the combo treatment was calculated from curves showed in Figure 2 and Chou Index was calculated using the formula (D_1_/D_X1_) + (D_2_/D_X2_), in which D_1_ and D_2_ are the IC_50_ of 5FU and VNR in the combination treatment while D_X1_ and D_X2_ are the IC_50_ of the 5FU and VNR in the single treatment.

To investigate the mechanisms underlying the effects on cell proliferation of VNR and 5FU, alone or in combination, under STD or mCHT protocol, we examined cell cycle distribution pattern in MDA-MB-231. FACS analysis indicated a significant decrease of G0/G1 population in 5FU-, VNR- and combo-treated *vs.* untreated cells under STD protocol whereas a significant decrease was observed only in VNR-treated *vs.* untreated cells under mCHT protocol. A variable but significant increase in apoptotic cells treated with VNR either alone or in combination with 5FU *vs*. untreated cells, was observed both in standard and metronomic procedure (Supplementary Figure 1).

### 5FU and VNR can induce in TNBC cells either apoptosis alone or in parallel with autophagic cell death, depending upon their schedule of administration

**Figure 3:**
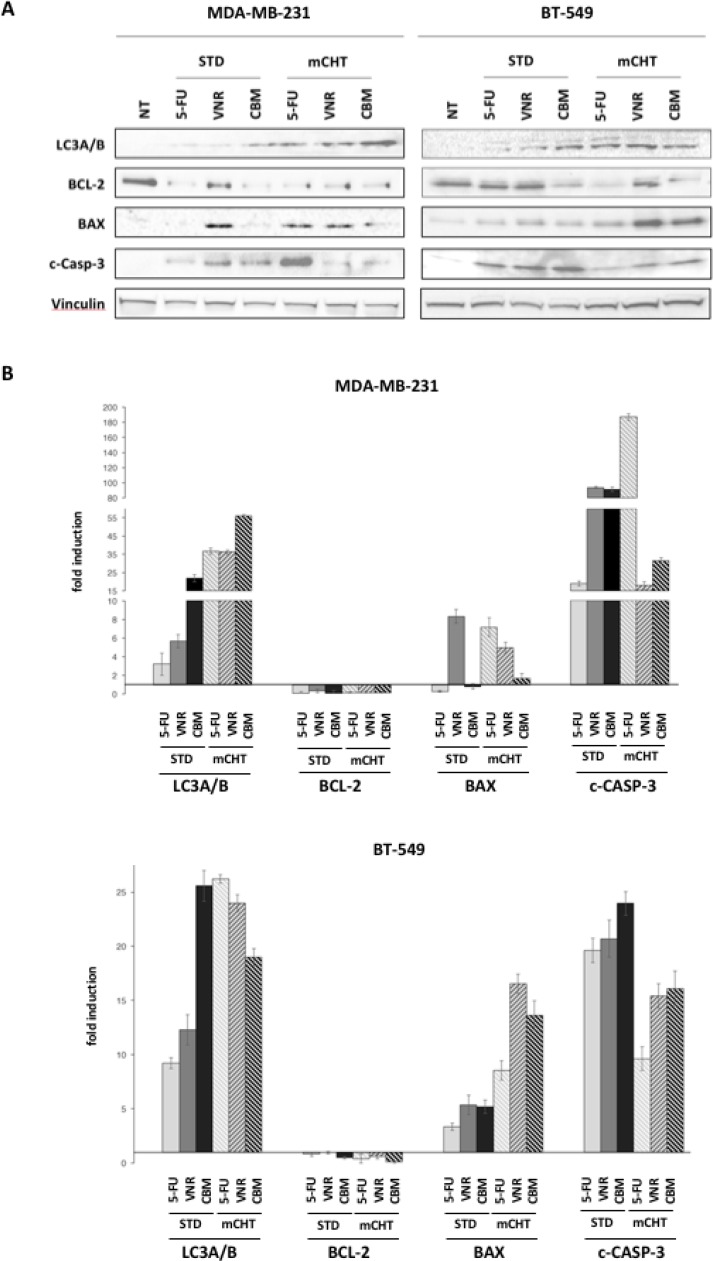
5FU and VNR can induce either apoptosis and/or autophagy in TNBC cells depending on the schedule of their administration (**A**) Upper panel: representative Western blot of MDA-MB-231 and BT-549 exposed to 5FU and VNR alone (IC_50_ single drug) or in combination (IC_50_ combo) for 4 h (STD) and for 96 h (mCHT). (**B**) Quantification of the protein expression as evaluated by densitometry. Protein levels were normalized to the corresponding Vinculin loading control. Error bars represent mean ± SEM, *n* = 3.

Recent studies have reported that activation of autophagy upon drug treatments can induce cell death either independently of or in parallel with apoptosis and necrosis [13]. To verify whether autophagy and/or apoptosis are triggered in cells treated with 5FU and VNR we assessed, by western blot, autophagic and apoptotic markers (Figure 3). In MDA-MB-231 and in BT-549 cells, upon exposure to 5FU and VNR alone or in combination in STD treatment, we observed an increased protein expression of microtubule-associated protein 1A/1B-light chain 3 (LC3A/B), a major constituent of the autophagosome that segregates the target protein/organelle and then fuses with lysosomes to form autolysosomes where the contents and LC3 are degraded [14–16]. A significant increase of LC3A/B expression was observed also upon exposure to 5FU and VNR, alone or in combination, under the mCHT schedule of treatment. These data indicate that the administration of 5FU and of VNR under mCHT protocol, in particular when they were given simultaneously, activated autophagy. Autophagy is interconnected with apoptosis by several molecular nodes of crosstalk, including Bcl-2/Bax and caspases [17]. We therefore examined the expression of the anti-apoptotic Bcl-2 protein and the pro-apoptotic Bax in MDA-MB-231 and in BT-549 cells treated with 5FU or VNR alone or their combination, under the two different protocols. We show that all these treatments, with the exception of 5FU and VNR in BT-549 cells under STD treatment, significantly decreased Bcl-2 protein expression compared to untreated cells. Furthermore, increased Bax expression was induced in BT-549 by all treatments which is consistent with induction of apoptosis. Interestingly, the increased expression of Bax correlated with up-regulation of cleaved caspase-3 expression when BT-549 cells were exposed to STD treatments, and to a lesser extent, under mCHT regimen. In the case of MDA-MB-231 up-regulation of cleaved caspase-3 clearly correlate with Bax induction, even though to a different extent, upon VNR exposure (under both, STD and mCHT, schedules) and when 5FU was given mCHT. In all other cases cleaved caspase-3 levels were increased in absence of Bax induction. Notably, cleaved caspase-3 levels are significantly lower in both cell lines under mCHT schedule (but for 5FU-treated MDA-MB-231 cells) compared to STD treatments. These results suggest that treatments with 5FU and VNR can induce either apoptosis alone or in parallel with autophagy in MDA-MB-231 and in BT-549 cells depending upon their schedule of administration.

### Metronomic treatment of MDA-MB-231 and BT-549 cells resulted in induction of autophagy

To corroborate the evidence of an autophagic process induced by 5FU and VNR in TNBC cells we immunostained treated cells with an antibody specific for LC3.

**Figure 4:**
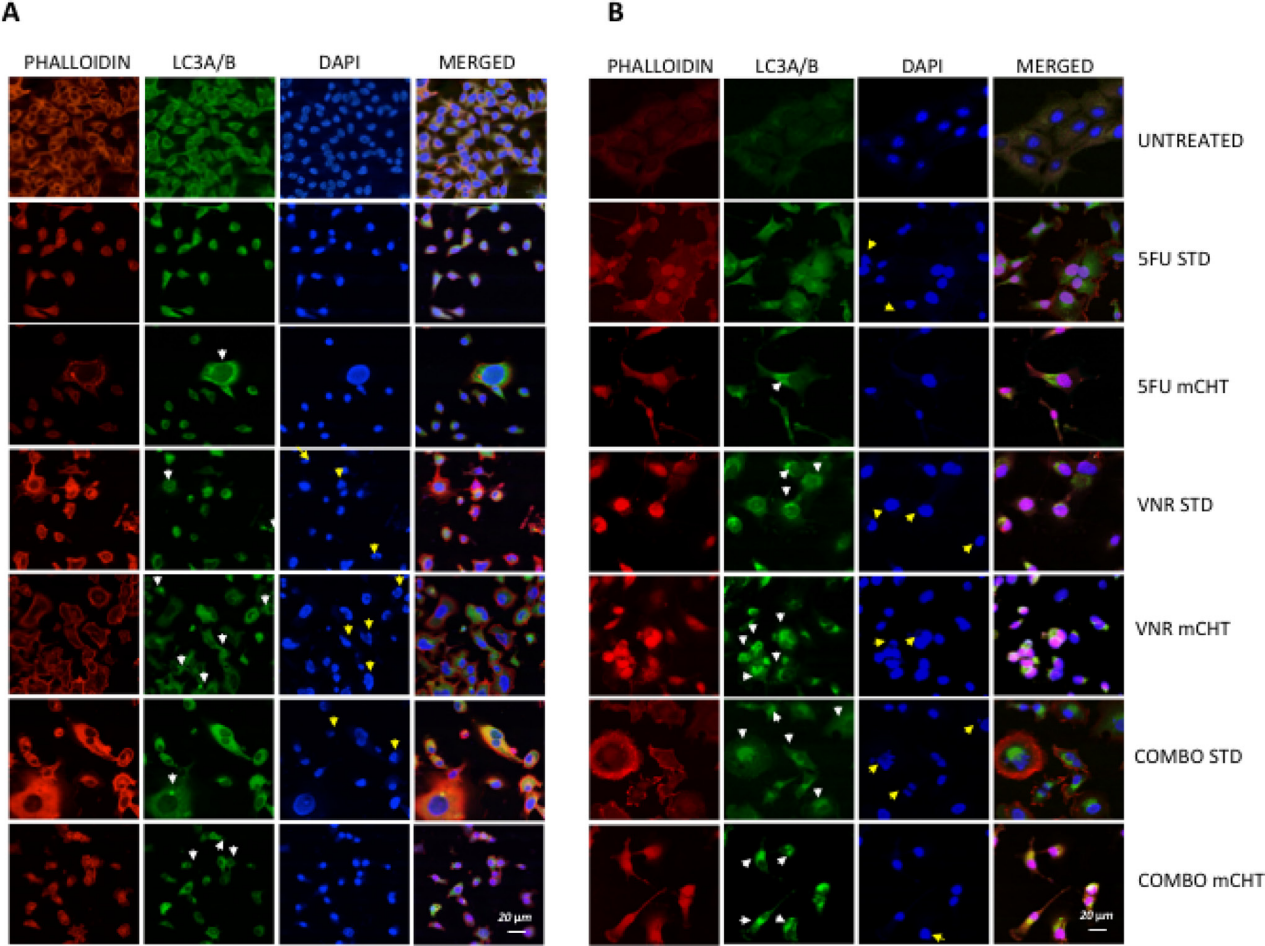
Increased of autophagy response in MDA-MB-231 and BT-549 cells treated with 5FU and VNR in metronomic schedule (**A**) MDA-MB-231 and (**B**) BT-549 cells were exposed to 5FU and VNR alone (IC_50_ single drug) or in combination (IC_50_ combo) for 4 h (STD) and for 96 h (mCHT). Anti-LC3A/B was detected by a FITC-conjugated secondary antibody, TRITC-conjugated phalloidin was used to stain actin and nuclei were counterstained with DAPI before acquiring images by confocal microscope (Biorad Laboratories, Hercules CA, USA). W*hite arrows* indicate autophagosomes fully formed in the cytoplasm of the cells upon exposure to VNR alone or in combination with 5FU in metronomic schedule. The *Yellow arrows* indicate apoptotic cells that are shrunken with condensed cytoplasm.

Untreated cells showed perinuclear actin and tubulin filaments uniformly distributed along the perimeter of the cell. VNR and 5FU, either alone and in combination, caused a rearrangement of the cytoskeleton. Moreover, in all three metronomic schedules of treatment (5FU, VNR and 5FU + VNR) there was an increase in LC3-positive punctate dots in perinuclear and cytoplasmic region, (pointed out by white arrows in Figure 4) indicating that induction of autophagy is dependent from the schedule of treatment of 5FU and VNR. Indeed, the formation of autophagosomes was not evident in cells under standard schedule of treatment, where only a weak, diffuse presence of the cytoplasmic form of LC-3 was detected. In addition, apoptotic cells that are shrunken with condensed cytoplasm (indicated by yellow arrows in Figure 4) were observed upon DAPI staining in cells treated with VNR under both STD and mCHT schedule.

DNA damaging drugs, including 5FU, have been shown to induce, besides apoptosis and autophagy, also cellular senescence [18, 19]. To reconcile the fact that in MDA-MB-231 cells mCHT administration of combined therapy seems less effective than STD administration in inducing both apoptosis and autophagy and to provide a possible mechanistic basis of the observed growth suppressive effect we tested SA-β-gal-activity (a marker of senescence) after treating the cells with 5FU and VNR, alone or in combination, under STD or mCHT protocols. Rare or few senescent cells were found after STD administration of 5FU and VNR, respectively. At variance, a significant increase of senescent cells was observed after mCHT administration of VNR alone and in combination with 5FU (Supplementary Figure 2).

On the whole, these data indicate that induction of apoptosis, autophagy and senescence all contribute to the therapeutic effect of metronomic schedule.

## DISCUSSION

The rapid development of new therapeutic drugs that target specific molecular pathways involved in tumor cell proliferation or apoptosis offers an extraordinary prospect to achieve a very high degree of specificity associated with lower toxicity [20]. Indeed, molecularly targeted agents often diverge from traditional cytotoxic agents due to their administration schedules and routes, their toxicity profiles and their antitumor activity. For some aspects, the development of metronomic chemotherapy is quite similar to that of targeted agents since the tumor response to metronomic schedule is due not only to antiangiogenic and immune-stimulatory effect, but also to a direct anti-cancer activity and could therefore be considered a multi-target therapy itself in contrast to conventional MTD or STD dose [21]. In addition, the mechanism of action of some anticancer agents can significant differ when they are given metronomically or by conventional schedules as reported by Harstrick *et al.* [22] who compared short and long exposure of human cancer lines to 5FU. Their results show that the inhibition of thymidylate synthase, a key rate-limiting enzyme of DNA synthesis, became more important when the treatment was prolonged. Other chemotherapy agents can show different mechanisms of action when administered according to a metronomic or a STD schedule. Remarkably, in women with breast cancer, re-treatment with metronomic CAPE can lead to a response after standard CAPE dosing has failed [23].

These different mechanisms of action may be the result of different effects on cell death [24]. While anticancer drugs usually kill cancer cells via apoptosis, low-dose mCHT can induce different types of cell death. For instance, Cortes *et al.* [25] reported that low doses of actinomycin D inhibited proliferation and induced apoptosis *in vitro*, as well as tumor regression *in vivo*, in a p53-dependent manner in a model of subcutaneously implanted neuroblastoma. However, a pan-caspase inhibitor only partially inhibited cell death induction, suggesting that the treatment could activate an apoptosis-independent cell death pathway. Bocci *et al.* [4] reported that the combination of metronomic topotecan and pazopanib significantly enhanced antitumor activity compared to monotherapy with either drug and prolonged survival, even in the advanced metastatic survival setting, with a marked decrease in tumor vascularity, proliferative index, and the induction of apoptosis.

In breast cancer, among the drugs used as single agents, CAPE and VNR are those supported by the greater amount of data. CAPE is an orally administered fluoropyrimidine carbamate, which was approved by FDA as a single agent for metastatic breast cancer patients. VNR is a semi-synthetic vinca alkaloid, active in a variety of cancers [26]. In breast cancer, oral VNR has been widely studied in metronomic regimens, with encouraging results [21]; for example, in the recent clinical study Victor-2, that evaluated a new metronomic combination (mCHT) of CAPE and VNR in metastatic breast cancer patients, the efficacy and safety of the metronomic combination of both drugs in an unselected group of patients has been shown [27].

Data emerged so far from pre-clinical studies and *in vitro* models, performed in both the adjuvant and the metastatic setting [28, 29], indicate that the metronomic combination of two different drugs allows to use doses of the single drugs that are much lower than those required by both the standard schedule or the single-agent metronomic administration [10].

On the clinical side, a great number of Phase II studies have been published starting from mid-2000s, showing an increasing interest of clinicians in mCHT: among the 80 publications selected for the systematic literature analysis by Lien and colleagues, 21 trials covered the topic of breast cancer involving 1135 patients. The authors identified 107 treatment regimens with at least one metronomic drug, being Cetuximab, Capecitabine, etoposide and VNR the most frequently used. The mean Response Rate (RR) of the pooled treatment regimens was 26%, with a mean Disease Control Rate (DCR) of 56.3%. Median duration of response was 4.6 months on average. This systematic literature review, even if not focused on breast cancer patients and TNBC in particular, confirmed that severe side-effects are rare, being observed in less than 5% of patients and the treatment-associated fatalities are very rare (0.4%) too, despite the fact that most study patients had an advanced disease, often refractory to often multiple prior conventional systemic therapies.

Even if international guidelines suggest the use of sequential, single agent regimens for the treatment of advanced breast cancer patients, they recommend the choice of combination regimens particularly for advanced TNBC ones, thus recognizing for these patients a strong clinical need for more aggressive strategies.

More recent trials, some of them conducted as single institution pilot experiences, tested different and more active drugs, mainly VNR and CAPE, reporting percentages of ORRs of approximately 50% and “Complete Bed Rest” of 77–80%.

In the *in vitro* study, called Victor-0, we focused our attention on the effect of 5FU and VNR in TNBCs given that they represent an important clinical challenge because they do not respond to endocrine therapy or other available targeted agents and have a poor response to STD chemotherapy as well. The metastatic potential in TNBCs is similar to that of other breast cancer subtypes, but these tumors are associated with a shorter median time to relapse and death [10].

In particular, using MDA-MB-231 and BT-549 cells as a model, we sought to investigate the cellular and molecular effects of mCHT *vs.* STD schedule of administration of different combinations of 5FU and VNR, in an attempt to elucidate the underlying mechanisms of their antiproliferative activities. We found that the exposure of MDA-MB-231 and BT-549 cells to 5FU/VNR inhibited the growth of cells at nanomolar concentrations and induced expression of cell death modulators such as Bax and cleaved caspase-3 and of autophagy such as LC3A/B. Moreover, we observed increased activity of SA-β-gal in cells treated with 5FU/VNR under mCHT. To investigate the mechanisms involved in 5FU- and VNR-induced growth suppression, we carried out flow cytometric analysis. Biziota *et al.* [30], reported that these drug concentrations did not have an obvious effect on the cell cycle. In our cellular model metronomic treatment showed lesser modification on cell cycle than STD administration of drugs. Conversely, the percentage of cell death increased significantly after treatment with VNR in both standard and metronomic schedules and also when cells were under exposure of both 5FU and VNR. These results suggest that VNR alone on in combination with 5FU induces cytotoxic effect on MDA-MB-231. A recent study has reported that activation of autophagy upon drug treatment can induce cell death either independently of or in parallel with apoptosis [13]. Autophagic cell death is mainly a morphologic definition (i.e. cell death associated with autophagosomes/autolysosomes), therefore, to understand whether autophagy was involved in the death of MDA-MB-231 and BT-549 cells treated with 5FU and VNR, we evaluated the punctate pattern of distribution of LC3A/B, structural proteins of the autophagosomal membranes widely used as biomarkers of autophagy. In all three mCHT schedules of treatment (5FU, VNR and 5FU + VNR) LC3A/B puncta signal was observed in the perinuclear region and throughout the cytoplasm. STD instead caused a strong rearrangement of the cytoskeleton not associated with the LC3A/B clustering typical of autophagosome membrane formation. In addition, we also observed that VNR induces apoptosis cell death either in STD or mCHT schedule of treatment in both cell lines. Even though morphological studies cannot prove a causative relationship between the autophagic process and cell death they are suggestive of a correlation between the two phenomena.

Shimizu S *et al.* [31] indicated that cytotoxic stimuli activate autophagic death in cells that are protected against apoptosis, such as those expressing antiapoptotic Bcl-2 or those lacking both Bax and Bak. In our *in vitro* mCHT *vs* STD models we observed that all treatments decreased Bcl-2 protein expression compared to untreated cells. Furthermore, increased Bax expression was elicited by all treatments which is consistent with induction of apoptotic cell death. Indeed, the increased expression of Bax correlated with up-regulation of cleaved caspase-3 levels in 5FU mCHT treatment whilst in VNR mCHT and in combo mCHT we observed a lesser increase of cleaved caspase-3 in both cell lines. We also evaluated whether treatments with 5FU and VNR could modulate LC3A/B expression in MDA-MB-231 and BT-549 cell lines. We found a significant increase in LC3A/B expression in cells upon exposure to 5FU and VNR alone that was further increased after co-exposure to 5FU and VNR under mCHT. Finally, we observed induction of cell senescence upon exposure to 5FU and VNR under mCHT way of administration. Cellular senescence is traditionally considered as a tumor-suppressive mechanism that irreversibly blocks cellular proliferation in response to a variety of stresses such as DNA damage, telomere attrition or cancer therapy [32]. Nevertheless, senescent cells have also been shown to promote neoplastic transformation and their autophagy-mediated elimination has been found to delay tumor growth [33]. In our study we found that cells treated with VNR alone or in combination with 5FU under mCHT express high levels of LC3A/B, are SA-β-gal positive and show low levels of cleaved casp-3 suggesting that autophagy and cellular senescence contribute more than apoptosis to the growth suppressive effect triggered by metronomic therapy. On the other hand, the major contribution to the growth suppressive effect on the STD regimen seems to rely on the induction of apoptosis and to a lesser extent to the triggering of autophagy. Thus, our results indicate that depending on the modality of administration of chemotherapy, TNBC cells respond differently by favoring senescence/autophagy vs apoptosis or concomitant induction of both. The findings that the activation of senescence, autophagy, and apoptosis are dose- and schedule-dependent and that these processes contribute to a different extent to the therapeutic outcome are particularly relevant taking into account that cancer cells are frequently defective at or become resistant to apoptosis: in these cases, triggering cellular senescence and autophagy could represent an alternative pathway to suppress cell growth and promote cell death. Accordingly, it has been shown that several anticancer agents induce autophagic cell death in cell lines and animal models and finally promote tumor regression [32, 34].

In conclusion, our data give novel insights and help to understand which molecular mechanism involved in the cell death of TNBC are triggered by the different chemotherapeutic treatments and/or schedules, even though much remains to be discovered in terms of the cross-talk between signals that mediate autophagy and apoptosis [35]. Therefore, improving our understanding of the mechanisms and relationships between conventional drugs, metronomic chemotherapy, and autophagy in the clinical setting is an important research topic. Such an approach will allow us to develop novel anticancer treatments that target signal transduction pathways related to cancer cell death.

## MATERIALS AND METHODS

### Materials

Human breast cancer cell line, MDA-MB-231 was purchased from American Type Culture Collection (ATCC, VA, USA). BT-549 cell were a kind gift from Dr. Luisa Lanfrancone (European Institute of Oncology, Milano, Italy). Cells were maintained in RPMI-1640 medium (Gibco, NY, USA), supplemented with 10% fetal bovine serum (FBS, Gibco), 100 units/ml penicillin and 100 mg/ml streptomycin (Euroclone). The cultures were incubated at 37°C in a humidified atmosphere with 5% CO_2_. Cells were passaged every 2–3 days to obtain exponential growth. Cells were cultured for a maximum of 4 weeks before thawing fresh, early passage cells. Cells were routinely tested for the presence of Mycoplasma by Hoechst stain.

5FU (Fluorouracil Teva^®^) was from San Gerardo Hospital (Monza, Italy). Vinorelbine ditartrate salt hydrate (VNR) was purchased from Sigma-Aldrich and then resuspended in DMSO following the manufacturer instructions. Methyl thiazolyl tetrazolium (MTT) was purchased from Sigma-Aldrich.

### MTT assay

Cell viability was assessed using the MTT assay. MDA-MB-231 and BT-549 cells were plated at a density of 1500 cells/well in 96-well plates [36]. The following day, the medium was replaced with growth medium containing either 5FU (10–200000 nM) or VNR (0.1–1000 nM). In the 96-h experiment, to simulate the metronomic dosing protocol, we replaced with fresh medium containing the appropriate drug concentration every 24 h, as reported by Biziota *et al.* [37]. To simulate the conventional administration protocol, which included exposure of MDA-MB-231 and BT-549 to 5FU or VNR for 4 h, with a washout period with drug-free medium for consecutive 92 h.

At the indicated time points, 1 mg/ml MTT was added to each well and cells were incubated for 3 hours. Then, plates were centrifuged at 2000 rpm for 10 minutes and cells were lysed with 100% Ethanol. The absorbance of formazane salt was measured at 540 nm using Infinite 200 Pro microplate reader (Tecan).

Results were expressed as a percentage of control cell proliferation and IC_50_ values were determined using Prism6 software (GraphPad Software Inc., La Jolla, California, USA).

### Drugs combination studies

The IC_50_ values obtained from single-drug cell viability assays were used to design subsequent drug combination experiments. We added to cells drugs at the following concentrations: 1) 2× IC_50_ (5FU) + 2× IC_50_ (VNR); 2) IC_50_ (5FU) + IC_50_ (VNR); 3) ½ IC_50_ (5FU) + ½ IC_50_ (VNR); 4) ¼ IC_50_ (5FU) + ¼ IC_50_ (VNR). The results obtained in the MTT assay were analyzed for synergistic, additive, or antagonistic effects using the combination index (CI) method of Chou-Talalay [12]. A CI was then determined using the equation: (D)_1_/(D_x_)_1_+ (D)_2_/(D_x_)_2_ which indicates that for x% inhibition of the dose, D_x_, the combined additive effect for the sum of the fractional doses of each drug, (D)_1_/(D)_2_ and (D)_2_/(D_x_)_2_ should be equal to unity. Instead, when CI < 1 the interaction is considered synergistic, when CI > 1 indicated antagonism.

Three independent experiments were performed with eight replicates per condition.

### Flow cytometry

For flow cytometry analyses, MDA-MB-231 cells were seeded in 100 mm dishes at a density of 500000 cells/dish. The day after, cells were serum starved for 24 hours. Then, cells were treated with the IC_50_ concentrations of VNR and/or 5FU under metronomic and conventional protocols. At 96 hours of incubation, cells were detached by trypsinization and 2 × 10^6^ cells were fixed with cold 70% Ethanol for 30 minutes. Then cells were washed with PBS and incubated with 20 ug/ml Propidium Iodide (Sigma Aldrich) and 0.2 mg/ml RNAse A (Life Technology) for 2 hours. Propidium Iodide incorporation was analysed with a FACSaria flow cytometer (Becton Dickinson).

### Immunofluorescence

MDA-MB-231 and BT-549 cells were plated in eight-chamber slide (Nunc) at a density of 2000 cells/well. The day after, cells were treated with the IC_50_ concentrations of VNR and/or 5FU under metronomic and conventional protocols. At 96 hours of incubation, cells were washed with PBS and fixed with 1:1 methanol-acetone (Sigma Aldrich) for 10 minutes at –20°C. Then, cells were washed three times with PBS and blocked with 3% BSA in TBS-Tween at room temperature for 1 hour. Cells were incubated at 4°C overnight with the following primary antibodies: anti-LC3A/B XP Rabbit mAb (1:100 Cell Signaling). The day after, cells were washed with 0.1% Triton x-100 TBS and beta actin was stained by incubation with Alexa fluor 555 Phalloidin (1:150; Invitrogen). After washing with PBS, nuclei were stained with DAPI (Sigma Aldrich). Stained cells were imaged by using confocal microscope (Biorad Laboratories, Hercules CA, USA) equipped with a Krypton/Argon laser and a red laser diode. Noise reduction was achieved by “Kalman filtering” during acquisition. All experiments were repeated at least three times and representative micrographs are shown in the Figures.

### Western blotting

MDA-MB-231 and BT-549 cells were seeded in 100mm dishes at a density of 500000 cells/dish. The following day, cells were treated with the IC_50_ concentrations of VNR and/or 5FU under metronomic and conventional protocols for 96 hours as described above. Cells were trypsinized and lysed in ice-cold RIPA buffer supplemented with 5 μg/ml aprotinin, 5 μg/ml leupeptin and 1 mM phenyl methyl sulphonyl fluoride. Protein concentration was measured by the BCA method (Sigma Aldrich). 30 μg of proteins were loaded and electrophoresed through 4–20% Tris-Gylcine gels in Tris-Glycine running buffer (all were Novex, San Diego, USA) for 2 hr at 100 volts. Then proteins were transferred on nitrocellulose membrane by using iBlot system (Invitrogen) following manufacturer's instructions. Membranes were blocked with 5% milk solution for 1 hour and incubated at 4°C overnight with the following primary antibodies: monoclonal rabbit anti-LC3A/B XP (1:1000 Cell Signaling), monoclonal mouse anti-human Bcl-2 (1:500, Cell Signaling), polyclonal rabbit anti-human Bax Ab (1:500, Santa Cruz), monoclonal mouse anti-human cleaved-Caspase 3 5A1E Cell Signaling (1:500, Zymed, CA, USA), Vinculin (1:5000, Sigma-Aldrich). After three washes with 0.05% PBS Tween, membranes were incubated at room temperature for 1 h with appropriate secondary antibody diluted in 5% nonfat dry milk in TBST. After three washing with 0.05% PBS-Tween, membranes were incubated with “Pierce™ ECL Western Blotting Substrate” for 5 minutes and proteins were detected using G:BOX Chemi System device (*SynGene*; Cambridge, UK). Immunoblotting was repeated at least three times with consistent results.

### Senescence-associated β-galactosidase activity assay

Senescence-associated β-galactosidase (SA-β-gal) activity was measured with a β-galactosidase staining kit (Sigma- Aldrich) according to the manufacturer's protocol. Briefly, cells were treated with the IC_50_ concentrations of VNR and/or 5FU under metronomic and conventional protocols as described above. After 96 hours TNBC cells were washed with PBS, fixed and stained with the β-galactosidase reagent. Cells were incubated overnight at 37°C without CO_2_ and then observed under a microscope. SA-β-gal positive blue-stained cells were counted per each observed field and the results reported as mean of percentages of cells per treatments (magnification 60X).

### Statistical analyses

Results were indicated as mean ± standard error of the mean (S.E.M.) or mean ± standard deviation (S.D.). Statistical analysis was performed using SPSS 17 software. Student's *t*-test was used to compare data between two treatment groups. Differences between more than two experimental groups were determined with one-way analysis of variance (ANOVA), and when significant differences among groups were found, a *post hoc* analysis (Tukey test) was used. A value of *p* < 0.05 was considered statistically significant.

## SUPPLEMENTARY MATERIALS FIGURES


