# Supplementary material for: Metronomic combination of Vinorelbine and 5Fluorouracil is able to inhibit triple-negative breast cancer cells. Results from the proof-of-concept VICTOR-0 study
Source: Oncotarget. 2018 Jun 8;9(44):27448–59. doi: 10.18632/oncotarget.25422 (PMC6007943; doi:10.18632/oncotarget.25422)
Supplement: Supplementary file 1 [file oncotarget-09-27448-s001.pdf]

Metronomic combination of vinorelbine and 5Fluorouracil inhibit triple-negativebreast cancer cells results from the proof-of-concept VICTOR-0 study

SUPPLEMENTARY MATERIALS

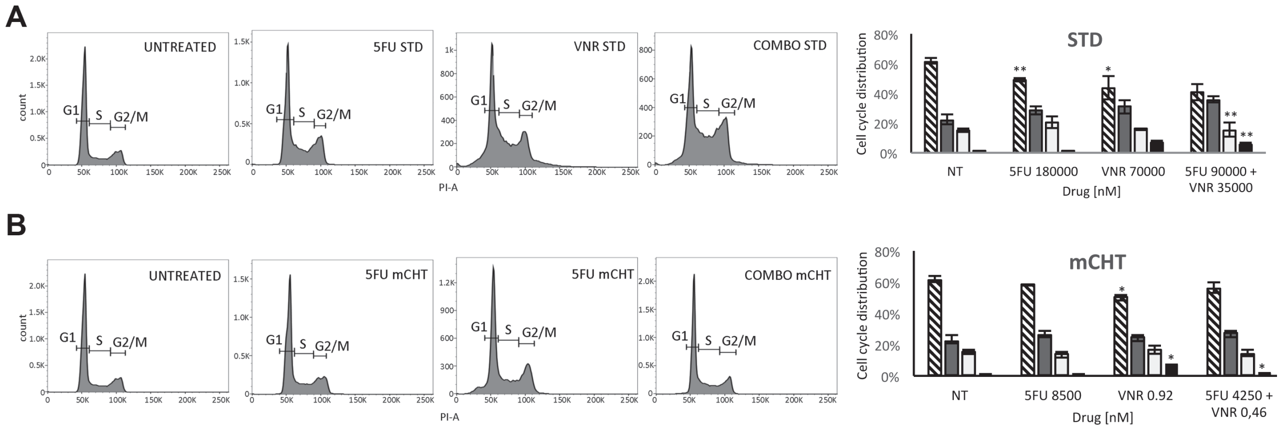

Supplementary Figure 1: Changes in cell cycle phase distribution following 5FU and VNR treatment. (**A**) MDA-MB-231 cells were seeded in DMEM supplemented with 10% fetal calf serum, and 24 h before treatment were synchronized in the cell cycle by serum starvation. Cells in serum-free medium were exposed to 5FU and VNR alone or in combination for 4 h (STD regimen) or 96 h (mCHT regimen) before cell cycle analysis was performed by FACS after propidium iodide DNA staining. (**B**) Cell cycle distribution after STD or mCHT treatment with 5-FU and VNR alone or in combination. $^{*}p < 0.05$ *vs.* untreated (NT), $^{**}p < 0.01$ *vs.* untreated (NT).

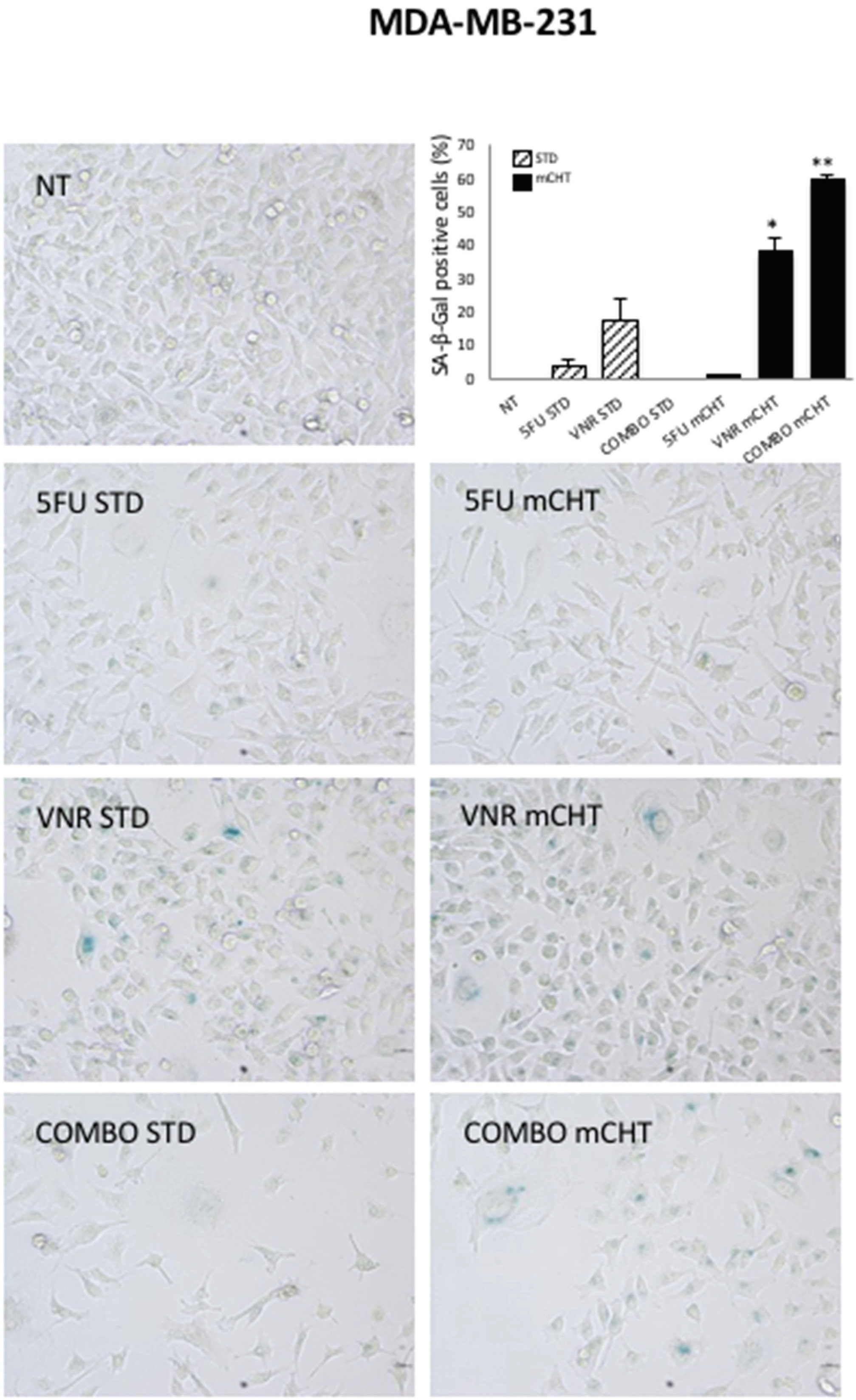

Supplementary Figure 2: Metronomic administration of 5FU and VNR induced a significant increase of the SA-β-Gal positive MDA-MB-231 cells. (**A**) Representative images of SA-β-GAL-stained blue-senescent MDA-MB-231 cells treated with 5FU and VNR alone (IC_{50} single drug) or in combination (IC_{50} combo) for 4 h (STD) and for 96 h (mCHT). (**B**) The ratio between the SA-β-galactosidase positive blue-stained cells and total nuclei was calculated for each observed field and the results are reported as the percentages of senescent cells per treatment. Error bars represent mean ± SEM, $n = 3$. $^{*}p < 0.05$ *vs* VNR STD, $^{**}p < 0.01$ vs COMBO STD.
